# Comparisons of seven algorithms for pathway analysis using the WTCCC Crohn's Disease dataset

**DOI:** 10.1186/1756-0500-4-386

**Published:** 2011-10-07

**Authors:** Hongsheng Gui, Miaoxin Li, Pak C Sham, Stacey S Cherny

**Affiliations:** 1 Department of Psychiatry, The University of Hong Kong, Hong Kong, SAR, China; 2 The State Key Laboratory of Brain and Cognitive Sciences, The University of Hong Kong, Hong Kong, SAR, China

## Abstract

**Background:**

Though rooted in genomic expression studies, pathway analysis for genome-wide association studies (GWAS) has gained increasing popularity, since it has the potential to discover hidden disease pathogenic mechanisms by combining statistical methods with biological knowledge. Generally, algorithms or programs proposed recently can be categorized by different types of input data, null hypothesis or counts of analysis stages. Due to complexity caused by SNP, gene and pathway relationships, re-sampling strategies like permutation are always utilized to derive an empirical distribution for test statistics for evaluating the significance of candidate pathways. However, evaluation of these algorithms on real GWAS datasets and real biological pathway databases needs to be addressed before we apply them widely with confidence.

**Findings:**

Two algorithms which use summary statistics from GWAS as input were implemented in KGG, a novel and user-friendly software tool for GWAS pathway analysis. Comparisons of these two algorithms as well as the other five selected algorithms were conducted by analyzing the WTCCC Crohn's Disease dataset utilizing the MsigDB canonical pathways. As a result of using permutation to obtain empirical p-value, most of these methods could control Type I error rate well, although some are conservative. However, the methods varied greatly in terms of power and running time, with the PLINK truncated set-based test being the most powerful and KGG being the fastest.

**Conclusions:**

Raw data-based algorithms, such as those implemented in PLINK, are preferable for GWAS pathway analysis as long as computational capacity is available. It may be worthwhile to apply two or more pathway analysis algorithms on the same GWAS dataset, since the methods differ greatly in their outputs and might provide complementary findings for the studied complex disease.

## Background

Simple single-marker tests used in genome-wide association studies (GWAS) have contributed to the discovery of many loci responsible for the variation observed in complex traits or disorders [1-3]; nevertheless, they are also criticized for their stringent significance threshold [4] and disregard of prior knowledge, which might lead to Type II errors, that is, not detecting real effects. Recently, a number of complementary approaches have been developed to prioritize susceptibility genes of complex diseases and increase power, of which meta-analysis, epistasis analysis and GWAS pathway analysis (GWASPA) are typical ones and already widely applied [5].

Biological pathways, which are actually series of actions among molecules in a cell that lead to a certain product or a change in a cell [6], are usually identified by experimental approaches [7,8] and then revised with bioinformatical mining tools [9]. Shared across different organisms, pathways play an important role in metabolism, gene regulation and signal transduction [10]. With genetic or environmental perturbations, some normal pathways might become dysfunctional and then contribute to complex diseases [11]. Pathway analysis for complex disease, also known as gene-set analysis, originated with genomic expression studies [12]; one of the two classical methods in that field is over/under representation analysis by hypergeometric test, while the other is gene-set enrichment analysis (GSEA) using Kolmogorov-Smirnov-like test statistics [13,14]. Since Wang et al. [15] first applied GSEA to GWAS data, more and more algorithms for performing genome-wide pathway analysis for SNP-chip datasets have emerged [16-19]. The PLINK set-based test utilizes average test statistics of groups of independent and/or truncated SNPs to provide a pathway-level test [17]; gene set ridge regression in association studies (GRASS) assesses joint association of pre-selected Eigen-SNPs for each gene in a candidate pathway with disease [18]; improved GSEA and association list Gene-ontology (GO) annotator (ALIGATOR) are two algorithms which utilize SNP-level test statistics or p-values in order to reduce computation cost over raw data-based methods [19,20]. These different algorithms could be categorized according to type of input data (raw data or summary statistics), counts of analysis stages (one or two stage), or basic hypothetical tests (competitive or self-contained) [16]. The difference between GWAS pathway analysis and classical pathway analysis in genomic expression studies lies in whether a gene-based score is directly provided or not. Previous research on multi-allelic association tests or gene-based tests, which always produce gene-level scores indirectly, provide a good bridge for pathway analysis on individual SNPs [21-23]. Among them, GATES is a rapid and powerful procedure for getting gene-based statistics which sometimes serve as a prerequisite for further advanced analysis [22].

Though several methods have already been proposed, there is no consensus as to the best method for conducting a GWASPA, especially when the underlying causal mechanism for disease at the functional level is not yet clear [16,24]. Results discrepancies among different methods on the same dataset might arise due to different mapping strategies from SNPs to pathways, or an algorithm's power of detecting susceptibility pathways, as well as incompatible pathway databases used [24]. Wang et al. [16] discussed the basic issues, main procedures and challenges involved in method development for GWASPA, but did not give a guideline of how to apply these methods from a practical perspective. Chen et al. [18] did compare the performance of different methods when evaluating the GRASS algorithm, but the simulation scenarios were designed for only one candidate pathway, and therefore need to be extended by examining both comprehensive simulated and real datasets on a genome-wide scale. Ballard et al. [25] illustrated the advantage of the random set method over the hypergeometric test for pathway analysis by analyzing three GWAS samples for Crohn's Disease (CD); however, they did not include any summary statistics-based algorithms which are becoming more and more popular [26].

As one of those complex diseases investigated at the earlier stage of GWAS [27], evidence for disease-causal variants, genes or even pathways related to CD are increasingly provided [3,25,27-29]. Meanwhile, the CD dataset from the Wellcome Trust Case Control Consortium (WTCCC) [30], which is openly available, has been repeatedly utilized and extensively explored by a variety of approaches [29,31,32]. This relatively abundant knowledge makes the WTCCC CD dataset a good testing sample for evaluating the performance of newly developed methods. Therefore, in this study, we implemented two summary statistics-based algorithms in an open-source tool named Knowledge-Based Mining System for Genome-Wide Genetic Studies (KGG) [22]; and then compared the performance of these two methods with another five existing methods using the WTCCC CD dataset, to evaluate the characteristics of the various methods, including Type 1 error, power and running time.

## Methods

### Algorithms

KGG is an open-source Java package developed for whole genome gene-based analysis, pathway analysis and protein-protein network analysis. Currently, it contains two classical algorithms for performing downstream pathway analysis after getting gene-level statistics by GATES [22], a novel gene-based method previously implemented in KGG. Formula 1 showed core idea of GATES, while formula 2 and 3 were basics of Simes' test [33] and hypergeometric test [34]; KGG would finally produce corresponding pathway-level results after running GATES-Simes or GATES-Hyper.

(1) $$P_{G}=\underset{j}{min}\{\frac{m_{e}P_{\left(j\right)}}{m_{e}{}_{\left(j\right)}}\}$$

Note: P _(j) _are the ordered j^th ^p-values (j from 1 to M) of the individual SNPs mapped to gene G; m_e _is the effective number of independent SNP p-values among all M SNPs, after accounting for the LD structure among these M SNPs; m_e(j) _is the effective number of independent SNP p-values among the top j SNPs, after accounting for the LD structure among these j SNPs.

(2) $$P_{GATES-sime(A)}=\underset{i}{min}\{\frac{kP_{G}{}_{\left(i\right)}}{i}\}$$

Note: Assume k genes mapped to pathway A, P_G (i) _calculated by GATES, is the i^th ^ordered gene p-value (i from 1 to k) among all k genes in pathway A.

(3) $$P_{GATES-Hyper\left(A\right)}=1-\overset{q-1}{\underset{i=0}{\sum }}\frac{C_{Q}^{i}C_{N-Q}^{n-i}}{C_{N}^{n}}$$

Note: N is the total number of genes in the whole gene list; Q is the number of those N genes in the pathway A; n is the total number of genes passing gene p-value threshold; and q is number of genes in pathway A out of those n genes.

Among the other five selected algorithms, only Aligator takes SNP-level summary statistics as input as GATES-Simes and GATES-Hyper does; for Aligator, a gene is treated as significant if it contains at least one SNP with p-value below predefined threshold. Each pathway is then tested for whether it contains more significant genes than expected by chance [20]. GSEAforGWAS selects the maximum SNP-level test statistic to represent a gene-level score, and then applies a weighted Kolmogorov-Smirnov running sum of competitive pathways to evaluate whether a particular pathway is enriched with top ranking genes or not [15]. GRASS calculates gene scores by combining regularized beta coefficients of pre-selected Eigen SNPs, following by getting the pathway-level score from standardized gene-level scores [18]. Both PLINK-Ave and PLINK-Max adopt the idea of a "set-based test", which computes a pathway level score directly from averaging SNP-level test statistics. After pruning SNPs in high LD, PLINK-Max only uses the top SNP in a pathway, but PLINK-Ave selects a few SNPs (default setting is up to the top 5 SNPs with p-value smaller than predefined threshold) [17]. All of the above five algorithms need to evaluate significance for pathway association with disease by permutation, and we permuted 1000 times for all methods.

### Evaluation data

880 canonical pathways (originated from KEGG [35], BioCarta [36] and Reactome [37]) which been manually curated by biology experts, were collected from the MsigDB database [38]. In comparison with Gene Ontology (GO) [39], which collects more broad functional categories in a hierarchical pattern, these canonical pathways represent relatively well-defined known biological pathways [40]. To further reduce pathway-level heterogeneity, we only included pathways which contained between 10 and 300 genes, which filtered out 27 pathways. Another potential CD causal pathway "IL12-IL23" [16,29] which was not in the database was also included, thus making the total number of selected candidate pathways to be 854. A survey of pathway size distribution, gene membership and overlapping ratio per pair-wise pathways was conducted by simple calculation using the 'Table' function in R. The WTCCC Crohn's Disease GWAS dataset was downloaded from the WTCCC website. Adopting the same quality control procedures as in the flagship publication [27], we included 1,748 CD patients as cases and 1,480 healthy individuals from the 1958 Birth Cohort [27] as controls. All the samples were genotyped with the Affymetrix 500 K chips. In total, 391,422 SNPs remained after quality control procedures for markers (minor allele frequency (MAF) > 0.05, Hardy-Weinberg equilibrium (HWE) p > 0.001, etc). Further selection of SNPs in or near (within 5 kb upstream or downstream) genes in the 854 canonical pathways resulted in 61,340 SNPs for subsequent pathway analysis. In order to run those summary statistics-based algorithms, we used PLINK (logistic regression model) to calculate SNP-level summary statistics (beta coefficient, odds ratio, p-value, etc.) with a general genomic control as population stratification adjustment. Due to lack of benchmark pathways for Crohn's Disease, we chose a subset of pathways from the 854 by enrichment analysis with GeneTrial [41], setting 93 candidate genes [3] previously reported for CD as input. This subset of pathways which were potentially associated with Crohn's disease was then recorded as list 1.

### Comparison of performance

We created a permuted dataset by assigning randomly shuffled case/control labels to original genotypes from the WTCCC CD dataset. Four algorithms (PLINK-AVE, PLINK-MAX, GSEAforGWAS and GRASS) were adopted to perform pathway analysis on this raw permuted data when the other three algorithms (GATES-Simes, GATES-Hyper and Aligator) on SNP level summary statistics from logistic regression analysis of raw simulated data. Each algorithm would produce a set of pathway level p-values. Since the null hypotheses assumed that no pathways were enriched by disease-susceptibility SNPs or genes for this simulated dataset, we estimated Type I error for each algorithm empirically by calculating the proportion of pathways with nominal p-values smaller than the critical threshold (set at 0.05) out of all 854 pathways. One sample Kolmogorov-Smirnov tests were performed to investigate whether observed pathway p-values followed a (0, 1) uniform distribution or not. Then algorithms which had an appropriate type I error rate were applied to the original CD dataset so as to prioritize potential causal pathways. In order to conduct these comparisons fairly, the same value was set when a predefined threshold was needed. False discovery rates (FDR) [42] were computed from pathway p-values produced by different algorithms, and pathways with FDR smaller than 0.05 were treated as significantly associated with CD and then marked as pathway list 2. Hypergeometric tests were conducted to check whether the number of overlapping pathways between list 1 and list 2 was greater than expected by chance. Nominal p-values and FDR for the IL12-IL23 pathway from different algorithms were also recorded and compared. Therefore, power of detecting real associated pathways for all 7 algorithms could be quantified by three indices: number of significant pathways, p-values from the hypergeometric test, and significance level for IL12-IL23 pathway. In addition, impact of varying p-value threshold and LD pruning cut-off on power were investigated with GATES-Hyper and PLINK-AVE, since such impact for other algorithms was either not necessary or addressed before [16,18,20].

## Results

### Survey on MsigDB canonical pathways

Table 1 presents characteristics of the 854 selected candidate pathways. Most of these pathways contain between 10 and 100 genes. Overlapping genes among pairs of pathways was less than 1 percent, allowing us to assume that the pathways were effectively independent, as required for GWASPA. However, investigation of genes mapping to pathways (also Table 1) showed that less than 30% of total pathway-included genes were unique to one pathway, while a few genes can even be covered by more than 100 different pathways. Pathway enrichment analysis for 93 candidate genes of CD revealed that 28 candidate pathways might be involved in causation of this disease (Additional File 1: Table S1). Literature evidence supported that 18 of these 28 pathways have good potential to associate with CD (also see Additional File 1: Table S1), though not all validated by experiments yet.

**Table 1:** Summary of selected Canonical pathways

Pathway size by gene		Pathway overlapping		Gene mapping to pathway	
Range	Proportion	Range	Proportion^1 ^	Range	Proportion
> = 10, < 20	36%	0	84%	Unique	26.20%
> = 20, < 100	55%	> 0, < 0.01	2.10%	> = 2, < 10	58.50%
> = 100, < 200	7.70%	> 0.01, < 0.1	11.30%	> = 10, < 100	15.10%
> = 200, < 300	1.30%	> 0.1, < 0.5	2.50%	> = 100	0.20%
		> 0.5	0.10%		

Note 1: consider all possible pairs from of 854 canonical pathways; define the ratio as sharing count of genes out of all different genes for one pair of pathways (union overlap).

A survey on 854 selected candidate pathways was conducted in order to investigate the characteristics of pathway size, pair-wise pathway overlap and gene allocation. Pathway size was measured by number of genes contained in the pathway; pathway overlapping was defined by the union overlapping ratio for one pair of pathways; gene allocation was counted as the number of pathways the genes belong to.

### Type I error rate comparison

**Figure 1:**
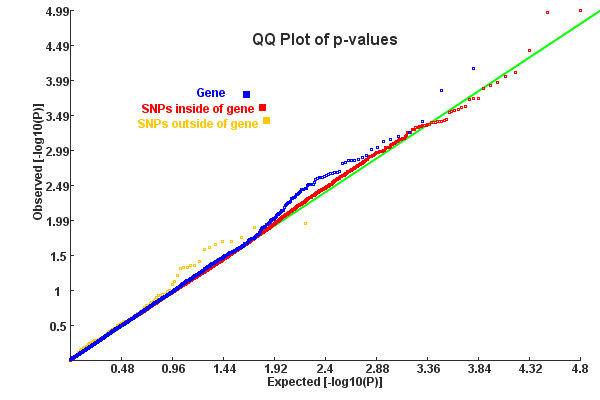
**QQ plot for SNP/gene p-values of a permuted CD dataset**. SNPs were divided into inside-of-genes and outside-of-genes according to their physical coordinates on the hg18 genome. Gene p-values were calculated by KGG, using the GATES algorithm.

**Figure 2:**
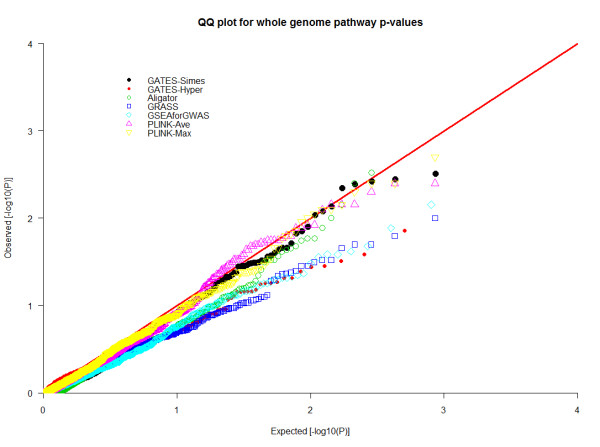
**QQ-plots for GWAS pathway p-values of seven algorithms for permuted datasets**. P-values for all 854 candidate pathways produced by each algorithm were plotted against their expected values from a (0, 1) uniform distribution.

Quantile-quantile (QQ) plots [43] for original SNP p-values and GATES-produced gene p-values in a permutated dataset are shown in Figure 1. No SNPs or genes mapping to selected pathways were apparently deviated from the theoretical straight line of a uniform distribution for this permuted dataset, indicating that the tests behave correctly under the null and the permutation procedure has eliminated all effects. Table 2 contains estimates of Type I error (at family wise error rate 0.05) for seven different GWASPA algorithms. Most of them were conservative since Type I error rates were below 0.05, two even smaller than 0.02 (GRASS, GATES-Hyper). This tendency was verified by one sample K-S test for (0, 1) uniform distribution (also Table 2) and QQ plot for pathway p-values (Figure 2).

**Table 2:** Type I error rate for seven algorithms

Algorithms	Type I error (0.05)	K-S test (two-sided)
GATES-Simes	0.043	< 2.2e-12
GATES-Hyper	0.016	5.4e-09
Aligator	0.032	2.6e-3
GRASS	0.018	5.1e-12
GSEAforGWAS	0.016	< 2.2e-16
PLINK-Ave	0.055	3.1e-06
PLINK-Max	0.036	9.5e-05

Two indices (Type I error and K-S test) were used to check whether those algorithms produced more false positive results than by chance. Type I error was calculated as proportion of pathways with nominal p-values < 0.05. The two-sided Kolmogorov-Smirnov test was used to investigate whether p-values from each algorithm follow the theoretical (0, 1) uniform distribution.

### Power and CD susceptibility pathways

**Figure 3:**
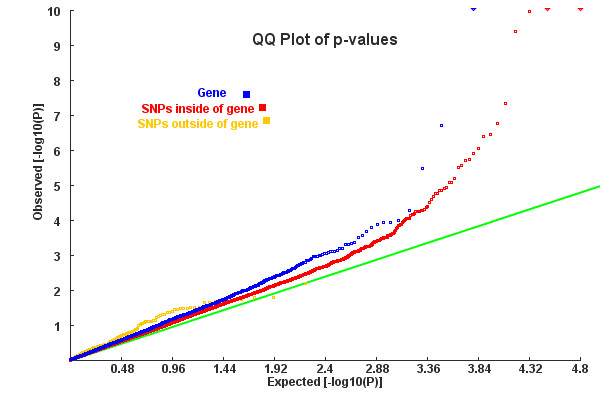
**QQ plot for SNP/gene p-values from the CD dataset**. SNPs were divided into inside-of-genes and outside-of-genes according to their physical coordinates on hg18 genome. Gene p-values were calculated by KGG, using the GATES algorithm.

A QQ plot of SNP-based test statistics from the WTCCC Crohn's Disease dataset shows that a bunch of individual SNPs and a few genes were significantly associated with CD (Figure 3). Table 3 presents three indicators of power of different algorithms for detecting hidden susceptibility pathways. Consistently, PLINK-Ave appears to be the most powerful algorithm, as it produced more significant pathways that overlapped with previously known pathways than any other algorithm (Table 3). However, it takes more consideration when running PLINK-Ave, which is affected by flexible setting of LD and p-value truncation cut-offs (Additional File 1: Table S2). In general, summary statistics-based algorithms (GATES-Hyper, GATES-Simes and Aligator) had less power than those raw data-based algorithms, and use of average statistics (PLINK-Ave and GRASS) was more powerful than relying on the top statistics within a given pathway (PLINK-Max and GSEAforGWAS). Only one pathway was detected in common by PLINK-Ave, GRASS and GSEAforGWAS, but there might be around 80 pathways in total possibly related to CD (Additional File 1: Table S3).

**Table 3:** Power indication from CD dataset

	**All candidate pathways**			**IL12-IL23 pathway**	
**Algorithms**	**No. of sign^1^**	**No. of known^2^**	**Hyper-test^3^**	**p-value**	**FDR**
		
GATES-Simes	4	3	1.20e-4	0.009	0.154
GATES-Hyper	0	0	--	0.005	0.493
Aligator	4	2	0.006	0.595	1
GRASS	41	3	0.146	0.031	0.158
GSEAforGWAS	10	2	0.04	0.004	0.118
PLINK-Ave	40	8	1.77e-5	0.002	0.043
PLINK-Max	0	0	--	0.006	0.155

Notes: 1, significance was defined as FDR for individual pathway smaller than 0.05; 2, no. of overlapping pathways between significant pathways in this study and previous known pathways for CD; 3, hyper-geometric test.

### Summary of running time and computing platform

Table 4 presents a summary of running times for all seven algorithms. Generally, algorithms with summary statistics as input were much faster than those utilizing raw data. Pathway analysis by GATES-Simes and GATES-Hyper could complete within one hour on a typical desktop computer. The time spent on computation was only several minutes as integrating marker LD information by KGG took most of the hour. Aligator and GRASS are both implemented in the R-SNPath package on a multi-core cluster, taking advantage of parallel computation. GSEAforGWAS was executed by the GenGen program following suggestion for parallel computation on their website--distributing permutations on four different nodes (each 250 times).

**Table 4:** Running time summary

Algorithm	Software	Input	Null Hypothesis	Computer configuration	Runtime
GATES-Simes	KGG^1^	Summary statistics	Self contained	Intel Core 2 Quad CPU Q9400 2.67 GHz, 4 GB RAM (desktop computer)	30 mins^5^
GATES-Hyper	KGG	Summary statistics	Self contained	As above	30 mins^5^
Aligator	R-SNPath^2^	Summary statistics	Competitive	Intel XEON 2 six-core x5670 2.93 Ghz, 128 GB RAM (cluster)	2 hours
GRASS	R-SNPath	Raw data	Self contained	As above	14 days
GSEAforGWAS	GenGen^3^	Raw data	Competitive	As above	2 days
PLINK-Ave	PLINK^4^	Raw data	Self contained	As above	40 hours
PLINK-Max	PLINK	Raw data	Self contained	As above	40 hours

Notes: 1, URL for KGG at http://bioinfo.hku.hk:13080/kggweb/home.htm; 2, URL for SNPath package at http://linchen.fhcrc.org/grass.html; 3, URL for GenGen program at http://www.openbioinformatics.org/gengen/; 4, URL for PLINK at http://pngu.mgh.harvard.edu/~purcell/plink/index.shtml; 5, excluding time spent building analysis genome (see KGG online manual).

## Discussion

It has been suggested that multiple genes in immune system functional pathways, especially those containing different Interleukin factors, might be involved in causation of Crohn's Disease [28,29]. Our findings of CD pathways across different algorithms also fall mostly into the category of immune response related pathways, though the number of significant pathways varied greatly for each individual algorithm. Likely, these differences are due to differences in four factors. The first is null hypothesis differences between self-contained tests and competitive tests [44,45]. PLINK-Ave, PLINK-Max, GRASS and GATES-Simes, which are self-contained tests (see table 4), assume that a pathway does not contain any significant SNPs or genes. But GATES-Hyper, Aligator and GSEAforGWAS are all based on competitive tests (see table 4), which aims to test whether genes in one pathway are enriched with a greater number of associated SNPs. The second contributor to the differences observed is differences in test statistic construction. PLINK-AVE and GRASS both applies an "average" concept which combines evidence of selected SNPs or genes to identify pathways with more overall association signal, but PLINK-Max, GATES-Simes, GATES-Hyper, Aligator and GSEAforGWAS all use a "maximum" concept when computing pathway score from SNP scores directly or indirectly, relying on the most associated SNP in a pathway. It is likely that the underlying causal mechanism for CD involves more pathways covering moderate effect SNPs than simply a few pathways with top SNP hits only [21], implying that many more SNPs than we can identify individually contribute to disease. A third contributor to the differences observed is whether genes are treated as an intermediate bridge from SNPs and pathways in a two-stage approach. Though it has been suggested that two stage pathway analysis was more robust and powerful than one stage pathway analysis [46], our analyses don't support this, with the PLINK set-based test found to be superior to other gene-based algorithms. Finally, different strategies of handling LD structure between SNPs can also explain part of the variation in performance. Pruning SNPs in high LD (PLINK), choosing Eigen SNPs by principal component analysis (GRASS and KGG), or focusing only on the top SNP (Aligator and GSEAforGWAS), are all effective ways of reducing inflation caused by SNP dependency, but may perform differently.

Due to complexity caused by SNP, gene, and pathway relationships, re-sampling strategies like permutation are always utilized to derive an empirical distribution for test statistics to evaluate the significance of candidate pathways. However, it comes with a cost of time and memory. Usually two approaches are used to lighten the computational burden, either a revised adaptive or optimal permutation of raw genotype/phenotype data [46,47] or a permutation of SNP or gene labels in summary statistics data [48]. We showed that GATES-Hyper and GATES-Simes implemented in KGG were much faster than those permutation-based algorithms since both of them used approximation for p-value distribution. Clearly, the advantages of GWASPA over genomic expression studies or traditional laboratory works (such as knock out mouse and cultured cells) [7,8] are apparent in terms of time and economy and can be used to guide these further studies.

There are also some limitations of our study. While the WTCCC CD dataset is an ideal model GWAS, the performance of GWASPA algorithms may not be as good with other datasets, where studies are smaller and less is known regarding function at the outset. We recommend applying two or more algorithms from different categories (self-contained versus competitive, average versus maximum and one-stage versus two-stage) in practice, especially when we cannot readily distinguish the scenario in which various susceptibility variants confer moderate risk to disease versus the scenario in which a major effect variant in a pathway plays a dominant role in complex diseases [15,20]. In addition, we set a fixed threshold for SNP or gene p-value truncation when including them for pathway analysis by PLINK-Ave, Aligator, and GATES-Hyper, but this cut-off is somewhat arbitrary and the choice made can influence results dramatically. Moreover, the FDR method was applied to correct for multiple testing and determine significance on pathway level, since almost no pathway p-values would survive Bonferroni adjustment [49]. Nevertheless, we think it was still reasonable for comparing performances of different algorithms, since we applied the same standards to all methods tested [24]. Lastly, none of these algorithms took differences in pathway structure stored in the original BioPAX or SBML format into consideration [50], but treated them as plain text containing independent gene symbols, when in fact any gene can be a member of multiple pathways. This drawback is handling by more advanced statistical methods like mixed-effect models and Bayesian networks, which facilitates modelling of gene-gene overlapping, interaction and correlation as well as net gene effect within the same pathway [51,52].

## Conclusions

GWAS pathway analysis, which prioritizes candidate pathways associated with complex disorders, could serve as an important complement to individual SNP analysis and gene-based analysis. Though all algorithms selected in this study do not have inflated Type I error rates, they vary greatly in terms of power and running time. The PLINK truncated set-based test was the most powerful, but the two summary statistics-based algorithms implemented in KGG were the fastest. However, raw data-based algorithms should be preferred for GWAS pathway analysis as long as computation capacity is available, since they preserve the intact data structure and tend to be more powerful than summary statistics-based algorithms. When underlying disease causal mechanism is ambiguous, which is common for complex diseases, it is worthwhile to apply two or more pathway analysis algorithms on the same GWAS dataset.

## Availability and Requirements

KGG is implemented using Java; therefore a Java Runtime Environment (JRE) is required to run KGG. Currently, installation of JRE and KGG are supported for Windows, Mac OS × and Linux. Three command files (run.win.bat, run.mac.sh and run.linux.sh) are provided for users to run KGG easily. A graphical user interface will automatically appear once initiating the command file. Documentation, source code, and precompiled binaries can be downloaded from http://bioinfo.hku.hk:13080/kggweb/home.htm.

## Competing interests

The authors declare that they have no competing interests.

## Authors' contributions

HG devised the study, performed simulation and data analysis, and drafted the manuscript. ML developed the KGG package and implemented GATES-Simes and GATES-Hyper algorithms. SC and PS contributed to study design and revised the manuscript. All authors read and approved the final manuscript.

## Supplementary Material

Additional file 1 **Potential CD-associated pathways by different methods**. **Table S1. Positive pathways for Crohn's disease from enrichment analysis of 93 CD susceptibility genes**. This table contains 28 enriched pathways (FDR < 0.05, hypergeometric test) from GeneTrail analysis. These pathways are treated as positive pathways for CD in this study. **Table S2. Number of significant pathways (FDR < 0.05) detected by PLINK-Ave and GATES-Hyper algorithm with different LD and p-value threshold setting**. This table shows impact of varying LD pruning and p-value threshold on detecting significant pathways for Crohn's disease. **Table S3. Overlapping between pathways detected by different raw data based algorithms**. This table presents significant pathways (FDR < 0.05) detected by PLINK-AVE, GRASS and GSEAforGWAS. The overlap between each pair of algorithms and also with positive pathways is illustrated by 'Yes' or 'No'. Click here for file
